# Red cell distribution width and platelet indices as predictors of mortality in acute coronary syndrome: A prospective observational study

**DOI:** 10.6026/973206300222592

**Published:** 2026-04-30

**Authors:** Hariom Gupta, Umesh Pratap Singh, Tripathi V.D, Subhash Shukla

**Affiliations:** 1 Department of General Medicine, Shyam Shah Medical College and Associated Sanjay Gandhi Memorial Hospital, Rewa, Madhya Pradesh, India; 2 Department of Cardiology, Super Specialty Hospital Block, Shyam Shah Medical College, Rewa, Madhya Pradesh, India

**Keywords:** ACS, red cell distribution width, mean-platelet volume, platelet distribution width, platelet count, cardiovascular outcome

## Abstract

Acute Coronary Syndrome (ACS) is a leading cause of cardiovascular mortality and simple hematological markers may help predict outcomes. Baseline laboratory parameters were recorded and patients were followed for in-hospital mortality and ICU requirement in this prospective study of 140 ACS patients admitted during 2024,. The mean age was 59.3 ± 10.7 years, with 65.7% males, and adverse outcomes occurred in 8.6%. Non-survivors had significantly higher RDW, mean platelet volume (MPV) and platelet distribution width (PDW). RDW-SD showed the highest predictive accuracy, followed by NLR and RDW-CV. Multivariate analysis identified creatinine, RDW, PDW, NLR, MPV, and urea as independent mortality predictors, while higher hemoglobin was protective, supporting the role of routine, low-cost markers in early ACS risk stratification.

## Background:

Acute Coronary Syndrome (ACS) is a spectrum of conditions resulting from reduced or blocked blood flow to the myocardium, encompassing unstable angina, NSTEMI, and STEMI [1]. It remains a major cause of global morbidity and mortality, highlighting the need for effective prognostic biomarkers to guide treatment strategies [2]. Recent evidence underscores the role of inflammation, oxidative stress, and endothelial dysfunction in ACS pathogenesis [3]. Hematological parameters such as Red Cell Distribution Width (RDW) and platelet indices have shown prognostic relevance. RDW, indicating red cell size variability, is linked to systemic inflammation and poor cardiovascular outcomes [4]. Platelets are central to atherothrombosis and thrombus formation [5] with indices like platelet count (PC), mean platelet volume (MPV), platelet distribution width (PDW), and plateletcrit (PCT) reflecting their activation status [6]. Elevated MPV and PDW are associated with increased thrombotic risk and ACS severity [7]. Studies suggest a correlation between altered RDW, platelet indices, and adverse clinical outcomes, including heart failure, arrhythmias, and mortality [8]. However, their predictive value across ACS subtypes remains under investigation [9]. The relationship between hematologic indices and established risk scores like GRACE has been demonstrated [10], with RDW showing particular utility in non-ST elevation ACS [11]. Blood count parameters provide cost-effective prognostic information [12], with RDW-to-erythrocyte count ratios offering additional predictive value [13]. Recent studies from diverse populations confirm these findings [14], while pathophysiological research continues to elucidate mechanisms linking atherosclerosis progression to hematologic changes [15]. Sex-specific differences in coronary risk factors may influence these relationships [16]. Admission platelet volume indices serve diagnostic and prognostic roles in acute chest pain presentations [17], with "real-world" analyses supporting RDW as an outcome predictor [18]. Therefore, it is of interest to evaluate RDW and platelet indices in ACS patients and correlate them with in-hospital mortality, potentially offering cost-effective tools for early risk stratification and management.

## Materials and Methods:

## Study design and setting:

This was a hospital-based, cross-sectional Prospective observational study conducted over a period of one year, from January 2024 to December 2024, in the Department of General Medicine at Sanjay Gandhi Memorial Hospital (SGMH) and the Department of Cardiology at the Super Specialty Block, both affiliated with Shyam Shah Medical College, Rewa, and Madhya Pradesh, India.

## Study population and sample size:

A total of 140 adult patients (aged ≥18 years) presenting with a confirmed diagnosis of Acute Coronary Syndrome (ACS) including ST- Elevation Myocardial Infarction (STEMI), Non-ST Elevation Myocardial Infarction (NSTEMI), and unstable angina, were enrolled in the study. The sample size was calculated using the formula n = 4pq/l^2^, considering the national prevalence of coronary artery disease (CAD) at 9.7%.

## Inclusion criteria:

Patients aged ≥18 years with a confirmed diagnosis of acute coronary syndrome (ACS), encompassing ST-elevation myocardial infarction (STEMI), non-ST-elevation myocardial infarction (NSTEMI), or unstable angina. The diagnosis must be established based on clinical features, characteristic electrocardiographic changes, and elevated cardiac biomarkers.

## Exclusion criteria:

Patients will be excluded if they have any of the following: known anemia or bleeding disorders; prior history of percutaneous transluminal coronary angioplasty (PTCA) or coronary artery bypass grafting (CABG); chronic kidney disease; chronic liver disease; valvular heart disease; pregnancy; recent blood transfusion; or platelet disorders or enzyme replacement therapy.

## Data collection:

After obtaining informed consent, demographic and clinical data were collected using a structured case record form. Blood samples were drawn prior to initiation of any pharmacologic therapy. Complete Blood Count (CBC) was performed and hematological parameters including Red Cell Distribution Width (RDW-CV and RDW-SD), Mean Platelet Volume (MPV), Platelet Distribution Width (PDW), Plateletcrit (PCT), Platelet Large Cell Count (PLCC), Platelet Large Cell Ratio (PLCR) and NLR were analyzed using a standardized automated hematology analyzer. Routine biochemical investigations including liver function tests (LFT), renal function tests (RFT), and lipid profiles were also performed.

## Outcome measures:

Patients were followed throughout their hospital stay. The primary outcomes recorded were in-hospital mortality and discharge status. The study primarily aimed to evaluate the association of RDW and platelet indices with clinical outcomes in ACS patients.

## Statistical analysis:

All statistical analyses were performed using SPSS software (version 2022). Quantitative variables were presented as mean ± standard deviation (SD), and categorical variables as frequencies and percentages. Comparison between groups was done using the independent t-test for continuous variables and chi-square test for categorical data. Receiver Operating Characteristic (ROC) curves was generated to assess the predictive ability of significant variables. A p-value < 0.05 was considered statistically significant.

## Results:

In this study comprising 140 patients with acute coronary syndrome (ACS), the mean age was 57.2 ± 11.66 years. As shown in Table 1 (see PDF), the age group most affected was >60 years (38.57%), followed by the 51-60 years group (34.28%), reflecting the typical age-related pattern of coronary artery disease. Males constituted a significantly larger proportion of the cohort (72.14%), highlighting the male preponderance in ACS presentation. Regarding residential distribution, 52.86% were from urban areas and 47.14% from rural areas, suggesting a nearly equal disease burden across regions. In terms of body mass index (BMI), no patients were classified as underweight or Obese Class II. The majority had a normal BMI (38.57%), while overweight and Obese Class I each comprised 30.71%, indicating that excess weight may play a substantial role in ACS pathogenesis. Tobacco chewing was the most prevalent form of addiction, observed in 61 patients (43.57%). Smoking was reported by 28 patients (20%), while alcohol consumption was noted in 19 patients (13.57%) (Table 1 - see PDF). Analysis of hematological parameters presented in Table 2 (see PDF) revealed that the mean neutrophil-to-lymphocyte ratio (NLR) was 5.17 ± 2.91, with the majority of patients (37.14%) falling into the >3-7 range, followed by 25.71% in the >7-11 range, suggesting an elevated inflammatory state in most cases. The RDW-SD was markedly elevated with a mean of 49.37 ± 9.28, and 27.85% of patients had values >56 fL, which may be linked to adverse outcomes. Similarly, RDW-CV was elevated (>14.5%) in 90% of patients, with a mean of 16.51 ± 2.94, reflecting significant anisocytosis and red cell size variability (Table 2 - see PDF). Platelet indices also showed notable changes. The mean MPV was 13.23 ± 1.69 fL, and 83.57% of patients had MPV >12 fL, indicating heightened platelet activation. PDW was elevated (>14) in 95% of patients, with a mean of 16.68 ± 1.52, further supporting enhanced platelet heterogeneity and reactivity (Table 2 - see PDF). The distribution of ACS types showed a predominance of STEMI (88.57%), while NSTEMI accounted for 10.71% and unstable angina was rare (0.71%). Most patients were discharged (91.43%), and the in-hospital mortality rate was 8.57%, as presented in Table 2 (see PDF). A comparative analysis of hematological parameters between STEMI and NSTEMI patients, as presented in Table 3 (see PDF), showed statistically significant differences across all evaluated markers. RDW-SD was notably higher in the STEMI group (45.78 ± 6.37 fL) compared to NSTEMI patients (42.73 ± 6.01 fL), with a p-value of 0.0403, indicating a significant association. Similarly, RDW-CV was significantly elevated in STEMI patients (16.26 ± 2.87%) versus NSTEMI (14.82 ± 0.77%), with a p-value of 0.0279. The neutrophil-to-lymphocyte ratio (NLR), a known inflammatory marker, was also considerably higher in STEMI cases (5.35 ± 2.93) than in NSTEMI (3.34 ± 1.80), achieving statistical significance (p = 0.0137). Moreover, mean platelet volume (MPV) was significantly greater in STEMI patients (13.15 ± 1.48 fL) than in NSTEMI (11.76 ± 1.71 fL), with a highly significant p-value of 0.0004. Platelet distribution width (PDW) was also elevated in STEMI (16.53 ± 2.01) compared to NSTEMI (15.08 ± 1.28), with a p-value of 0.0038. In this study, various hematological and biochemical parameters were analyzed between patients who were discharged and those who died during hospitalization (Table 4 - see PDF). Hemoglobin levels were significantly lower in the mortality group (11.97 ± 2.60 g/dL) compared to the discharged group (13.53 ± 1.83 g/dL), with a statistically significant p-value of 0.0037.

The red blood cell (RBC) count was also significantly lower among non-survivors (3.875 ± 0.84 million/µL vs. 4.25 ± 0.61 million/µL, p = 0.0251. Although total leukocyte count (TLC) was slightly elevated in patients who died (13.70 ± 5.38 x10^3^/µL) compared to survivors (12.82 ± 5.05 x10^3^/µL), the difference was not statistically significant (p = 0.2827). RDW-SD was markedly elevated in non-survivors (60.83 ± 4.67 fL vs. 45.25 ± 6.42 fL, p < 0.0001), indicating strong predictive value. RDW-CV also showed a significant difference (19.00 ± 0.58% vs. 16.00 ± 2.86%, p = 0.0002). The neutrophil-to-lymphocyte ratio (NLR) was significantly higher in the death group (10.40 ± 1.78) than in those discharged (5.15 ± 2.97, p < 0.0001), making it a valuable prognostic marker. Among platelet indices, mean platelet volume (MPV) and platelet distribution width (PDW) were significantly elevated in non-survivors (p = 0.0195 and p = 0.0254, respectively). In contrast, plateletcrit (PCT), platelet count (PLCC), and platelet large cell ratio (PLCR) did not differ significantly between the groups. Renal dysfunction was associated with mortality, as evidenced by higher serum creatinine (1.24 ± 0.48 mg/dL vs. 1.00 ± 0.22 mg/dL, p = 0.0008) and blood urea levels (42.63 ± 16.58 mg/dL vs. 29.86 ± 11.97 mg/dL, p = 0.0009) in the death group. Random blood sugar (RBS) levels were numerically higher in non-survivors (156.33 ± 63.89 mg/dL) compared to survivors (142.65 ± 58.71 mg/dL), but the difference was not statistically significant (p = 0.4223). Similarly, total cholesterol (TC), triglycerides (TG), and LDL values did not show statistically meaningful differences. Interestingly, HDL levels were significantly lower in the mortality group (40.29 ± 10.47mg/dL) versus the discharged group (53.27 ± 38.39 mg/dL, p = 0.0065), though the clinical significance of this finding requires further investigation. These findings are summarized in Table 4 (see PDF). Figure 1 (see PDF) presents ROC Curves for Predictors of Mortality in ACS Patients (Receiver Operating Characteristic (ROC) curves demonstrate the predictive accuracy of various hematological and biochemical markers for in-hospital mortality among patients with Acute Coronary Syndrome (ACS). RDW-SD showed the highest predictive power (AUC = 0.98), followed by NLR (AUC = 0.97), RDW-CV (AUC = 0.88), serum creatinine (AUC = 0.84), PDW (AUC = 0.73), and MPV (AUC = 0.69). The diagonal dashed line represents the line of no discrimination (AUC = 0.5).) The ROC curve analysis (Figure 1 - see PDF) evaluated the ability of various biomarkers to predict mortality in patients with acute coronary syndrome (ACS). As shown in Figure 1 (see PDF), among the parameters analyzed, red cell distribution width-standard deviation (RDW-SD) exhibited the highest discriminative power with an area under the curve (AUC) of 0.98, indicating excellent predictive performance. The neutrophil-to-lymphocyte ratio (NLR) also showed strong predictive ability with an AUC of 0.97. RDW-coefficient of variation (RDW-CV) and serum creatinine demonstrated good predictive accuracy, with AUCs of 0.88 and 0.84, respectively. In comparison, platelet distribution width (PDW) showed moderate predictive capability (AUC = 0.73), while mean platelet volume (MPV) had the lowest AUC of 0.69, reflecting limited predictive value. These findings highlight the potential role of RDW-SD, NLR, RDW-CV, and serum creatinine as useful biomarkers for risk stratification in ACS patients, with RDW-SD emerging as the most powerful individual predictor. Figure 2 (see PDF) presents Multivariate Logistic Regression Analysis for Predictors of Mortality in ACS Patients. Forest plot of multivariate logistic regression analysis illustrate the adjusted odds ratios (OR) and 95% confidence intervals (CI) for independent predictors of mortality in patients with acute coronary syndrome (ACS). Elevated levels of serum creatinine, RDW, PDW, NLR, MPV, and serum urea were associated with increased odds of mortality, whereas higher hemoglobin levels were associated with reduced mortality risk. The red dashed line indicates the reference line (OR = 1), representing no association. Figure 2 (see PDF) presents the results of the multivariate logistic regression analysis, identifying several independent predictors of mortality in patients with acute coronary syndrome (ACS). Elevated serum creatinine was associated with the highest odds of mortality (OR: 2.20; 95% CI: 2.00-2.40), followed by increased red cell distribution width (RDW) (OR: 2.00; 95% CI: 1.80-2.20), platelet distribution width (PDW) (OR: 1.80; 95% CI: 1.60-2.00), neutrophil-to-lymphocyte ratio (NLR) (OR: 1.60; 95% CI: 1.40-1.80), and mean platelet volume (MPV) (OR: 1.40; 95% CI: 1.20-1.60). Elevated serum urea levels also conferred a higher mortality risk (OR: 1.20; 95% CI: 1.00-1.40). In contrast, higher hemoglobin levels were significantly associated with a reduced risk of mortality (OR: 0.76; 95% CI: 0.60-0.95). These findings underscore the prognostic value of routine hematologic and biochemical parameters in the risk stratification of ACS patients.

## Discussion:

In this study of 140 acute coronary syndrome (ACS) patients, the majority were aged over 60 years (38.57%), with a mean age of 57.2 ± 11.66 years. This age distribution is consistent with prior studies by Acet *et al.* (2014) [8], Bekler *et al.* (2015) [9], Adam *et al.* (2018) [10], Xiao *et al.* (2020) [11], and Tadesse *et al.* (2024) [12], all of which emphasize the higher prevalence of ACS in older adults. A male predominance (72.14%) was observed, echoing findings from Tadesse *et al.* (2024) [12], Xiao *et al.* (2020) [11], and others, reflecting established epidemiological trends related to hormonal and lifestyle risk factors. Urban residents comprised 52.86% of patients, slightly higher than rural patients (47.14%), mirroring findings from Tadesse S *et al.* (2024) [12] that suggest urbanization-associated risk factors and better diagnostic access. Regarding substance use, 43.57% chewed tobacco, 20% smoked, and 13.57% consumed alcohol behaviors linked to atherosclerotic risk, as reported by Jebari-Benslaiman *et al.* (2022) [13] and Kim (2024) [14]. BMI analysis showed 38.57% of patients had normal BMI (18.5-22.9), while 61.42% were overweight or mildly obese, with a mean BMI of 23.92 ± 2.53. This aligns with a downward BMI trend seen in Dehghani *et al.* (2014) [15], Talarico *et al.* (2021) [16], and Tadesse *et al.* (2024) [12]. Despite normal BMI in many, ACS risk persists, emphasizing the importance of multifactorial risk profiling beyond body weight alone. In the present study involving 140 acute coronary syndrome (ACS) patients, key hematological and clinical biomarkers were evaluated for their diagnostic and prognostic relevance. The neutrophil-to-lymphocyte ratio (NLR) had a mean value of 5.17 ± 2.91, with most patients (62.85%) displaying values >3, indicating significant systemic inflammation. These findings are consistent with Tadesse *et al.* (2024) [12] and Li *et al.* (2024) [17], who reported NLR means of 4.98 and 10.4, respectively, and affirmed its role in predicting severe coronary lesions and mortality. Wang *et al.* (2023) [18] also confirmed rising NLR quartiles correlated with increased cardiovascular mortality The RDW-SD mean was 49.37 ± 9.27 fL, with 27.85% exceeding 56 fL, reflecting anisocytosis. This aligns with findings by Tenekecioglu *et al.* (2015) [19] and Raval *et al.* (2024) [20], who associated higher RDW-SD with myocardial injury and severe CAD. The RDW-CV in this study was also elevated (mean 16.51 ± 2.94), with 90% of patients >14.5%, a pattern mirrored in studies by Karahan *et al.* (2021) [21] and Talarico *et al.* (2021) [16], highlighting RDW-CV's predictive value for mortality.

Mean MPV was 13.23 ± 1.69, and 83.57% had values >12, denoting high platelet activation. Similar elevations were observed by Raval *et al.* (2024) [20] and Tenekecioglu *et al.* (2015) [19], supporting MPV's role in assessing thrombotic risk. The PDW mean was 16.68 ± 1.52, with 95% >14, confirming findings by Talarico *et al.* (2021) [16] and Wang *et al.* (2023) [18] who associated PDW with adverse cardiac outcomes. Clinically, STEMI was the most prevalent ACS type (88.57%), similar to distributions seen in studies by Ghafoor *et al.* (2024) [22] and Karahan *et al.* (2021) [21]. The in-hospital mortality rate in the current study was 8.57%, within the typical reported range of 5.8-9.1% as documented by Raval *et al.* (2024) [20], Wang *et al.* (2023) [18], and Talarico *et al.* (2021) [16]. Together, these findings underscore the utility of NLR, RDW, MPV, and PDW as accessible, cost-effective prognostic biomarkers in ACS management, reinforcing global evidence on their predictive validity. In the comparison between STEMI and NSTEMI patients, STEMI cases demonstrated significantly higher values of RDW-SD, RDW-CV, NLR, MPV, and PDW. These findings suggest greater anisocytosis, heightened systemic inflammation, and more pronounced platelet activation in STEMI, reflecting its more aggressive pathophysiology. These trends align with several published studies over the past decade. Tenekecioglu *et al.* (2015) [19], Karahan *et al.* (2021) [21], and Talarico *et al.* (2021) [16] reported elevated NLR, MPV, and PDW levels in STEMI patients, further reinforcing their diagnostic value. Crucially, when outcomes were analyzed, several biomarkers were found to differ significantly between deceased and discharged patients. RDW-SD and RDW-CV were markedly elevated in non-survivors, indicating increased anisocytosis linked to poor prognosis. Similarly, NLR, MPV, and PDW were significantly higher among patients who died, reflecting severe systemic inflammation and heightened platelet activation. Lower RBC and hemoglobin levels in non-survivors suggest that anemia may contribute to adverse outcomes. Additionally, elevated serum creatinine in deceased patients indicates a potential role of renal dysfunction as an independent risk factor. These associations are strongly supported by previous studies from Tenekecioglu *et al.* (2015) [19], Talarico *et al.* (2021) [16], Wang *et al.* (2023) [18], and Raval *et al.* (2024) [20]. Interestingly, other variables such as WBC count, total platelet count, PCT, PLCC, PLCR, RBS, Cholesterol, Triglyceride and LDL showed no statistically significant differences between survivors and non-survivors, indicating that not all routine hematological markers possess equal predictive power.

## Conclusion:

We show that RDW, NLR, MPV, PDW, hemoglobin, serum creatinine, and serum urea are reliable surrogate markers of inflammation, platelet activation, renal dysfunction, and systemic stress in acute coronary syndrome. While these parameters do not localize myocardial infarction, they correlate strongly with disease severity and mortality. Routine incorporation of these inexpensive and widely available markers can improve early risk stratification and support timely, targeted management in patients with ACS.

## Advancement of knowledge:

This study shows that simple hematological markers like RDW and platelet indices can effectively predict outcomes in Acute Coronary Syndrome. It highlights RDW-SD as a strong predictor, along with NLR and RDW-CV. The findings support the use of low-cost, routinely available parameters for early risk stratification in ACS patients.
